# Fecundity of Paternal and Maternal Non-Parental Female Relatives of Homosexual and Heterosexual Men

**DOI:** 10.1371/journal.pone.0051088

**Published:** 2012-12-05

**Authors:** Andrea Camperio Ciani, Elena Pellizzari

**Affiliations:** 1 Department of General Psychology, University of Padova, Padova, Italy; 2 GEA, International Association for the Study and Conservation of Ecosystems, Padova, Italy; Tor Vergata University of Rome, Italy

## Abstract

A variety of social, developmental, biological and genetic factors influence sexual orientation in males. Thus, several hypotheses have attempted to explain the sustenance of genetic factors that influence male homosexuality, despite decreased fecundity within the homosexuals. Kin selection, the existence of maternal effects and two forms of balancing selection, sexually antagonistic selection and overdominance, have been proposed as compensatory mechanisms for reduced homosexual fecundity. Here, we suggest that the empirical support for kin selection and maternal effects cannot account for the low universal frequency and stability of the distribution of homosexuals. To identify the responsible compensatory mechanism, we analyzed fecundity in 2,100 European female relatives, i.e., aunts and grandmothers, of either homosexual or heterosexual probands who were matched in terms of age, culture and sampling strategy. Female relatives were chosen to avoid the sampling bias of the fraternal birth order effect, which occurs when indirectly sampling mothers though their homosexual sons. We observed that the maternal aunts and grandmothers of homosexual probands were significantly more fecund compared with the maternal aunts and maternal grandmothers of the heterosexual probands. No difference in fecundity was observed in the paternal female lines (grandmothers or aunts) from either of the two proband groups. Moreover, due to the selective increase in maternal female fecundity, the total female fecundity was significantly higher in homosexual than heterosexual probands, thus compensating for the reduced fecundity of homosexuals. Altogether, these data support an X-linked multi-locus sexually antagonistic hypothesis rather than an autosomal multi-locus overdominance hypothesis.

## Introduction

The origin and causes of male homosexuality remain largely debated, and several mechanisms have been proposed to explain this condition. It has been suggested that the prenatal endocrine environment significantly influences human sexual orientation, and that biological factors, including genetic susceptibility, could potentially interact with postnatal social factors to determine life-long sexual orientation [1]. However, genetic evidence from family studies comparing adoptive brothers, biological brothers, and monozygotic twins have shown that the probability of homosexuality among brothers progressively increases, which strongly suggests a partial genetic influence [2], [3], [4], [5]. The evidence-based assumption of even a partial genetic predisposition for homosexuality in males generates evolutionary questions and presents several factors that contradict the Darwinian assumption that natural selection should progressively eliminate factors reducing individual fecundity and fitness. This contradiction results in a *Darwinian paradox* because homosexual men are less fecund and produce fewer offspring than heterosexual men. Thus, it is reasonable to conclude that genes influencing homosexuality would be eliminated from the population if not for the existence of a potential compensatory mechanism [6], [7]. In the last two decades, researchers have begun to clarify the male homosexual conundrum. Most studies have shown that homosexuality is commonly present in certain families [8], [9], [10], [11], [12]. Studies on homosexual brothers employing DNA linkage analyses suggest an increased rate of homosexuality in the maternal line [10]. This conclusion has prompted the comparison of X-chromosomes in brothers, which can only be maternally inherited. These studies have suggested that a putative genetic factor is located on the long arm of the X-chromosome in the q28 region [10], [13], [14]; however, subsequent studies have failed to fully support this gene mapping hypothesis [15]. However, the results of successive studies continually suggest a genetic influence on male homosexuality [16]. It is well accepted that homosexual males reproduce significantly less than their heterosexual counterparts [17], [18], [19].

Various avenues of research have suggested an array of potential compensatory mechanisms to resolve this evolutionary conundrum, including kin selection, maternal effects, and two types of balancing selection–sexually antagonistic selection and overdominance. To resolve the conundrum related to male homosexuality, there must be a genetic mechanism that compensates for the reduced fecundity of homosexuals and satisfies at least two fundamental conditions derived from empirical evidence: 1) *stability*, which refers to genetic factors that potentially influence male homosexuality, which never go extinct and are not transmitted to all of the males in any population, and 2) *low frequency*, which suggests that homosexuality exists at a relatively lower frequency compared with heterosexuality [20], [21].

### Fecundity Compensation via Kin Selection

In the early 1970s, Trivers and Wilson proposed the idea of fecundity compensation through kin selection [22], [23], suggesting that while subjects might not reproduce directly, increased fecundity in close relatives is promoted through direct support and assistance to close relatives who share genes, thus compensating for the lower reproductive rate observed among homosexuals. There is sufficient evidence from behavioral genetics that gene selection through direct support to kin is a powerful mechanism to enhance the transmission of genes from one generation to the next [24]. However, this genetic mechanism could not be confirmed for homosexuals in U.S. populations. Bobrow and Bailey observed that male homosexuals do not lend behavioral, emotional or financial support to close kin that would significantly enhance inclusive fitness, and social contact with both parents in some patterns even appears to be contrary [25]. However, subsequent evidence from a Samoan population has provided some support for kin selection. [26], [27], [28].

Unfortunately, further studies in both eastern and western industrialized societies have not provided evidence for kin selection [29], [30], [31] The cross-cultural variability in the kin-directed altruism of homosexual males is not a universal characteristic that could account for the evolution of the genetic influence of homosexuality within the human population and does not exclude the possibility that kin selection might coexist with other mechanisms; thus, this hypothesis *per se* cannot be considered to be a self-sufficient solution for the homosexual conundrum. Therefore, two potential mechanisms for balancing selection in male homosexuality have been explored: sexually antagonistic selection [11], [21] and overdominance [19], [32], [33].

### Sexually Antagonistic Selection

In 2004, Camperio Ciani et al. [11] suggested that male homosexuality was a by-product of a genetic factor that increases fecundity in females. This notion was based on data showing an increased frequency of homosexuality in the maternal line of homosexual probands [11],[34] and that both mothers and maternal aunts were more fecund in homosexual probands compared with heterosexual probands [11], [21], [34], [35], [36]. Additional evidence was found in support of the sexually antagonistic selection and female fecundity hypothesis independent of the fraternal birth order (*see the further fraternal birth order section for an introduction to this effect*). The responses from a large Internet survey also indicated that homosexual mothers exhibit higher fecundity compared with heterosexual mothers, independent of the fraternal birth order effect [37]. These results were later confirmed for maternal aunts in a wide Caucasian sample population [36] and were replicated in mothers of Samoan androphilic males, which have significantly more children than gynephilic males [38], [39]. However, these same authors recently showed that not only the maternal but also the paternal grandmothers of Samoan androphilic males (fa'afafine) produce more offspring than gynephilic males, which does not support the predictions of the X-chromosome linkage hypothesis [40].

Balancing selection via sexually antagonistic selection is a genetic mechanism based on a multi-locus genetic factor partially located on the X-chromosome, which could potentially promote androphilic behavior (attraction to males) in carriers. In males, this genetic factor increases the probability of homosexuality through androphilia, whereas in females, it increases fecundity through androphilia [11], [21], [34], [35], or better through a complex pattern of behavior, personality and enhanced fertility that increases attraction from males and fecundity [35]. According to the sexually antagonistic hypothesis, the localization of such a factor on the X-chromosome should increase fecundity only in maternal-line females sharing the X-chromosome with the homosexual subject and not in paternal-line females or any other related male. A mathematical analysis of the population dynamics has shown that this sexually antagonistic selection mechanism respects the empirical assumptions of both *stability* and *low frequency* [21].

### Overdominance

In 2005, King and colleagues [32] proposed different conclusions while investigating a large British clinical sample. These authors performed a different analysis on probands based on family size, rather than fecundity and concluded that all members of a homosexual family compensate with increased fecundity [32]. Contrary to the predictions of the X-chromosome linkage hypothesis, King et al. (2005) showed that, compared with heterosexual males, homosexual males had significantly more aunts, uncles and cousins in the paternal line, but not the maternal line [32]. Schwartz et al. (2010) showed that, compared with heterosexual males, the paternal grandmothers of homosexual males have higher offspring production, but not the maternal kin [19]. The observation of an enlarged family size in homosexual probands suggests a balancing selection mechanism through overdominance, which has since been suggested in other studies [19], [41], [42].

Overdominance suggests that a co-dominant genetic factor in autosomes promotes both fecundity and homosexuality. If this factor were present as a single copy (heterozygous) in an individual genome, an increase in fecundity would be observed, whereas if two copies (homozygous) of this factor were present, the probability of male homosexuality would increase and should not affect female fecundity. It is possible that a multi-locus genetic trait in males results in behavior that is more conducive to reproducing and supporting offspring among heterozygotes, and that this trait in the homozygotic condition might produce male homosexuality. The greater reproductive fitness or fecundity the biological relatives of homosexuals men would thus offset the selection pressure against homosexuality [33]. A mathematical analysis of population dynamics suggests that this mechanism, if based on at least two autosomal loci, respects both of the empirical assumptions of *stability* and *low frequency* [21]. Thus, if confirmed, such a mechanism could resolve the conundrum.

### Fraternal Birth Order Effect

Numerous studies have shown that the existence of older brothers increases the probability of homosexuality in later-born human males [43]. The probability of a male homosexuality increases with increasing numbers of older biological brothers, a phenomenon referred to as the fraternal birth order effect. The fraternal birth order effect has been reviewed several times [44], [45], [46]. Biological brothers increase the probability of homosexuality in later-born males, even if these siblings are reared in different households, in contrast to stepbrothers or adoptive brothers, which have no effect on sexual orientation [47].

The most likely explanation for the fraternal birth order effect is the progressive immunization of certain mothers to male-specific antigens through subsequent male fetuses and the increasing effects of anti-male antibodies on the sexual differentiation of the brain in successive male fetuses. These explanations do not necessarily suggest a direct genetic influence on sexual orientation but rather a potential maternal immune response to Y-linked histocompatibility antigens [46], [48]. Alternatively, it has been suggested that potential sexual contact with older brothers [49] or less rigid parenting might also potentially enable younger sons to more freely admit their sexuality [32]. However, Bogaert (2006) finding that the fraternal birth order effect only exists with biological brothers, contradict these alternative interpretations [47]. The maternal effect, defined as the fraternal birth order effect, has generally been examined in relation to the number of older brothers [43]. Blanchard [48] estimated that the population “risk” for homosexual orientation due to the fraternal birth order effect is approximately 33% for each older brother. Further support for the effect of fraternal birth order includes the much lower ratio of first-born to second- or third-born status among homosexual probands compared with the corresponding ratio in heterosexuals, particularly in low fecundity populations [11]. Few studies have reported that homosexual men also have greater numbers of older sisters than expected [32], [37], [38], [39], [50].

The fraternal birth order effect has now been observed in a large number of studies. Theoretically, however, the maternal effect cannot account for the conundrum. A systematic mathematical analysis of the propagation and equilibrium of putative genetic factors associated with male homosexuality in a population based on the use of the selection equation for one or two biallelic loci and Bayesian statistics for pedigree investigation showed that maternal effects violate the empirical requisite of both *stability* and *low frequency* [20], [21]. Thus, if the compensatory effect of homosexuality only results from a maternal effect (fraternal birth order) mechanism, then a rapid extinction or total diffusion of homosexuality in most populations would be observed. Both effects are inconsistent with historical and empirical observations. The persistence of male homosexuality in the human population has been confirmed through archaeological evidence, indicating that homosexuality occurred prehistorically and is therefore not a recent phenomenon [51], [52], [53], [54].

This finding does not suggest that a fraternal birth order effect does not occur, but rather that the fraternal birth order effect alone cannot explain the persistence of genetic factors that partially influence homosexuality associated with the low frequency of reduced fecundity in all populations. The same conclusion applies to other maternal effects, such as maternal genomic imprinting [21].

### Balancing Selection: Evidence from First-born Homosexuals and Younger Siblings

The identification of a balancing selective mechanism is complicated by the fact that fraternal birth order predicts an increase of older brothers due to a maternal effect in a family of homosexual probands. Thus, alternative balancing selection hypotheses should also show increased fecundity outside of the predictions of fraternal birth order. Increases in fecundity as an artifact (sampling bias) of the fraternal birth order effect are possible because randomly selected homosexual groups tend to include more older brothers than well-matched heterosexual comparison groups; consequently, homosexual groups exhibit larger sibships [42]. The sampling bias of fraternal birth order is observed when homosexuals are used as probands to investigate the fecundity of their mother. To clarify whether increases in fecundity resulting from a maternal effect are distinct from increases in fecundity that result from balancing selection, it is necessary to examine fecundity when the fraternal birth order effect is absent. The absence of the fraternal birth order effect can be observed through a comparison of the fecundity of mothers with homosexual firstborn sons, verifying the existence of a larger number of total offspring [11], [34], [41], [55] or comparing the number of younger siblings between similarly matched homosexual and heterosexual probands [37]. These data support balancing selection as a factor independent of maternal effects. An independent role of fecundity from the fraternal birth order effect has been suggested for high fertility populations, such as Samoa. [38] This question could also be addressed through a comparison of the fecundity of non-parental female relatives, particularly, individuals not selected for having a homosexual son and, thus, whose fecundity cannot be ascribed to the fraternal birth order effect, but notwithstanding should be more fecund according to the balancing selection hypothesis. Specifically, the fecundity of aunts and grandmothers, independent from having a homosexual male offspring is informative, as these individuals would not be selected from their own offspring and thus the fraternal birth order effect would not bias the sample toward an artificially larger family size and would not interfere with fecundity predictions. In this case, any differences in sibship size (averaging out religious, socioeconomic, ethnic, or other confounding cultural or demographic effects trough an adequate sampling design) could be reasonably attributed to differences in specific parental fertility.

### Research Question

The aims of this study are to determine the balancing selection mechanisms, if any, which allow the persistence of genetic factors that influence male homosexuality in the population. The fecundity of aunts and grandmothers of homosexual vs. heterosexual probands facilitates an analysis of three competing hypotheses with mutually exclusive predictions.

1. The lack of differences in either maternal or paternal female fecundity will provide strong empirical evidence that all fecundity variations in homosexuals are exclusively due to a side effect of fraternal birth order, as indirectly suggested [55].
2. Increased fecundity in both maternal females and paternal females will provide empirical evidence for overdominance as type of the balancing selection, as indirectly suggested [19], [32].
3. Increased fecundity in only the maternal line of female relatives of homosexual probands will provide evidence for sexually antagonistic selection as the type of balancing selection, as predicted [21].

## Methods

Here we analyzed the data obtained from all aunts and grandmothers, both maternal and paternal, of all probands collected in our laboratory of Evolutionary Psychology from 2002 to 2011. Portions of this sample were included in previous publications [11], [34], [35] but without consideration of age differences, which is now included. These data were extracted from the pedigrees of all probands, both homosexual and heterosexual, and included 2100 females: 955 aunts or grandmothers of heterosexuals and 1145 aunts or grandmothers of homosexuals. The data were collected in Northern Italy, Spain and France. The samples included only females for whom total fecundity had been definitively assessed, and all women were over the age of 50 at the time of sampling. The homosexual probands were sampled using the targeted sampling methodology of Watters and Biernacki [56] for accessing ‘hidden’ populations. To match for, religious, ethnic and socio-cultural variables that might influence the demography, the subjects were recruited from various associations for homosexual men, such as discotheques and beaches, and the heterosexual proband control group was sampled from after-work clubs, discotheques, and beaches located in the same geographical region, thus controlling for the ages of the subjects. Sampling was performed at different times to ensure that subjects with different habits were represented. The questionnaire was designed to acquire information on the sexual orientation of the proband. The sexual orientation was self-reported and confirmed through answers to five questions from the seven-point Kinsey scale addressing sexual self-identification, fantasy, attraction, imagination and personal behavior [57]. The data for the pedigree analysis were collected face-to-face, and according to the protocol, the questions were repeatedly cross checked and all efforts were made to precisely ascertain the family members fecundity, including the age of every female in the family of the proband, as previously described [11]. With the assistance of a researcher, the subjects was also asked to provide information regarding the age and the number of offspring of his maternal grandmother, including his mother, the age and number of offspring of his paternal grandmother including his father, and the individual age and number of offspring of each of his maternal and paternal aunts and uncles. The counts included individuals and/or offspring who were deceased, in which case we asked the age that these individuals would have been at the time of the interview and whether these individuals had any offspring. The information provided was considered to be trustworthy due to the anonymity of the questionnaire, its simplicity (e.g., recalling the numbers of only aunts, uncles and first cousins) and the lack of emotionally laden questions. For eleven percent of all aunts and twenty-nine percent of grandmothers, age could not be directly assessed either due to difficulties in recollection, or because these individuals were not well known to the proband. In these cases, the missing data were assigned the average age of the corresponding maternal or paternal female relative with the corresponding relationship (aunt or grandmother) to the proband. The distribution of all of the observed age data was reliable and did not differ from the average age of childbearing in Italian females (the mean age of all females of the corresponding cohort giving birth in that year), the average age difference within couples at marriage, and the average age of both paternal and maternal grandmothers [58], [59]. In accordance with ethical standards, the questionnaire was anonymous. The probands were informed that the scope of the research was “to investigate sexual orientation and fecundity in family members” and upon consent, the questionnaires were presented. No written consent was requested, as it was not compatible with the mandatory requirements of anonymity of the research. The subjects were also informed that they could withdraw from the questionnaire at anytime, and the questionnaire would be destroyed. No individuals refused the questionnaire once initiated. A total of 4% of all questionnaires were eliminated due to incomplete answers, e.g., not recalling the number or age of most female relatives.

The fecundity data are presented here as both raw data (Table 1, 2 and 3), ignoring the age distribution of females between the two samples, and subsequently covaried for age (Table 4 and 5). We present the covariance analysis for the sake of comparability with the data from previous researches [55], [41]. Considering that the sampling was obtained over a span of approximately ten years (from 2002 to 2011), each female in the sample was assigned an absolute age for 2012 to further control for cohort effects. The analysis of covariance assumes that the slopes of the separate regression lines for the two samples do not significantly differ and that the variance is homogeneous. These assumptions were both confirmed. No significant regression coefficient was observed for homosexual and heterosexual females. The significant Levine homogeneity test confirmed the homogeneity of the variances in our samples, thus facilitating the analysis of co-variance (ANCOVA) for age to normalize all of the cohort effects.

**Table 1:** Sample size and age differences.

	N	Average Age	SD	*t*	*p*
Homosexual	264	38.64	9.20	3.555	**.0001**
Heterosexual	240	40.07	8.77		
Homosexual maternal aunts	355	66.84	9.54	.625	.532
Heterosexual maternal aunts	284	67.31	8.94		
Homosexual paternal aunts	297	71.98	8.74	2.821	**.005**
Heterosexual paternal aunts	255	74.07	8.59		
Homosexual maternal grandmothers	246	94.39	9.30	1.882	.060
Heterosexual maternal grandmothers	208	95.99	8.75		
Homosexual paternal grandmothers	247	99.32	9.16	1.958	.051
Heterosexual paternal grandmothers	208	100.98	8.73		
Homosexual total female relatives	1145	91.10	16.70	2.178	**.030**
Heterosexuals total female relatives	955	82.69	16.68		

**Table 2:** Comparison of raw fecundity between females of the heterosexual and homosexual samples via *t*-tests.

	Heterosexuals			Homosexuals					
*Class of relatives*	N	Av. Fec.*	SD	N	Av. Fec.*	SD	df	*t*	*p*
Maternal aunts	280	1.54	1.07	347	1.98	1.26	625	4.588	**.000**
Paternal aunts	251	1.84	1.23	280	1.82	1.18	529	.182	.856
Maternal grandmothers	208	3.33	1.77	246	3.65	1.98	452	1.841	.066
Paternal grandmothers	208	3.36	2.03	245	3.32	1.75	451	.218	.827
Maternal aunts and maternal grandmothers	488	2.30	1.66	593	2.67	1.79	1079	3.482	**.001**
Paternal aunts and paternal grandmothers	459	2.53	1.81	525	2.52	1.65	982	.070	.944
All female relatives	947	2.41	1.74	1118	2.60	1.73	2063	−2.468	**.014**

* *Average fecundity.*

**Table 3:** Comparison of raw fecundity between paternal and maternal line females using *t*-tests.

	Maternal aunts			Paternal aunts					
	N	Av. Fec.*	SD	N	Av. Fec.*	SD	df	*t*	*p*
Heterosexuals	280	1.54	1.07	251	1.84	1.23	529	−2.929	**.004**
Homosexuals	347	1.98	1.26	280	1.82	1.18	625	1.635	.103
	**Maternal grandmothers**			**Paternal grandmothers**					
	**N**	**Av. Fec.**	**SD**	**N**	**Av. Fec**	**SD**	**df**	***t***	**p**
Heterosexuals	280	3.33	1.77	208	3.36	2.03	414	−.205	.838
Homosexuals	246	3.65	1.98	248	3.32	1.75	489	1.944	.**052**

* *Average fecundity*.

**Table 4:** ANCOVA of the fecundity of female relatives of heterosexuals and homosexuals, with individual age as a covariate controlling for cohort age differences.

	Heterosexuals		Homosexuals				
*Class of relatives*	N	Av. Fec.*	N	Av. Fec.*	df	*F*	*p*
Maternal aunts	280	1.54	347	1.96	624	21.283	**.000**
Paternal aunts	251	1.83	280	1.83	528	.003	.960
Maternal grandmothers	208	3.31	246	3.67	451	3.972	**.047**
Paternal grandmothers	208	3.47	245	3.42	450	.001	.977
Maternal aunts and maternal grandmothers	488	2.27	593	2.70	1078	19.98	**.000**
Paternal aunts and paternal grandmothers	459	2.48	525	2.55	981	.312	.576
All female relatives	947	2.38	1118	2.63	2062	13.087	.**000**

* Average fecundity.

**Table 5:** ANCOVA of the fecundity of maternal and paternal females with individual age as a covariate controlling for cohort age differences.

	Maternal aunts		Paternal aunts				
	N	Av. Fec.*	N	Av. Fec.*	df	F	*P*
Heterosexuals	280	1.56	251	1.82	528	5.543	**.019**
Homosexuals	347	2.00	280	1.79	624	4.265	.**039**
	**Maternal grandmothers**		**Paternal grandmothers**				
	**N**	**Av. Fec.** *	**N**	**Av. Fec.** *	**df**	**F**	***p***
Heterosexuals	208	3.38	208	3.30	413	.149	.700
Homosexuals	246	3.69	245	3.28	488	5.478	**.020**

* *Average fecundity*.

## Results

Table 1 shows that the distribution of the present sample sizes with respect to the average age of the interviewed subjects who reported their family fecundity were well matched (38.64 for homosexual probands and 40.07 for heterosexual probands), although the minimal difference between the two sample populations was significant (t = 3.555, p<0.001). The age difference between paternal aunts was also significant (t = 2.821 p = 0.05), with the paternal aunts of heterosexuals being slightly older. However, the largest age difference, of about 5 years, was found between the paternal and maternal relatives samples, which reflected the national habit of marrying male partners that are 5 years older on average. This age difference at marriage is relatively constant among cohorts from the 1950s to the present, with a slight decrease in recent years (ISTAT, 2010).

Table 2 shows that the maternal aunts of homosexuals were significantly more fecund than the corresponding aunts of heterosexuals in a raw analysis that did not consider age cohort effects. In addition, the maternal grandmothers of homosexuals were more fecund than the corresponding grandmothers of heterosexuals, although this difference was not significant in this raw data analysis (*t* = 1.84, *p* = 0.066). Altogether, these findings show that the maternal aunts and maternal grandmothers were significantly more fecund that the corresponding females in the heterosexual sample (*t* = 3.482 *p* = .001). In contrast, the fecundity of the paternal aunts and grandmothers of homosexuals, either alone or together, was not significantly different from that of the corresponding females in the heterosexual sample. Considering the entire sample together, all of the female relatives of the homosexual probands were significantly more fecund than the females from the heterosexual sample (*t* = 2.467 *p* = .014), reflecting superior maternal fecundity.

Table 3 presents the comparison of maternal versus paternal females among the female relatives of both homosexual and heterosexual probands. We observed that the paternal aunts of heterosexuals were significantly more fecund than their maternal aunts, whereas the opposite was observed for the aunts of homosexuals, whose maternal aunts were more fecund than the paternal aunts, although these results were not significant. While maternal grandmothers were more fecund than paternal grandmothers for homosexuals (t = 1.944 p = 0.052), with a marginal significance, there was no difference in grandmother fecundity in the heterosexual sample. These results were obtained using raw data with no correction for age differences.

In Tables 4 and 5, we show the difference in fecundity when the age differences and cohort fecundity effects were considered. Table 4 shows that considering age differences with an ANCOVA, the comparison of all of the maternal females indicates a significant increase in fecundity compared with heterosexual females, while none of the paternal female fecundity classes showed significant differences between homosexuals and heterosexuals. Thus, the total fecundity of homosexual females was significantly higher than that of the female relatives of heterosexuals.

Finally, Table 5 shows that when age differences were controlled via an ANCOVA, the paternal aunts of heterosexuals were more fecund than their maternal aunts, whereas the maternal aunts and maternal grandmothers of homosexuals were significantly more fecund than their paternal aunts and grandmothers.

## Discussion

The fecundity rates and differences between the groups examined in this study were indeed similar to those observed in previous studies detecting higher homosexual in the maternal line fecundity and no increase in paternal female fecundity [6], [11], [18], [21], [34], [35], [36]. In the present sample (Table 2), without controlling for age differences, all of the maternal female line fecundities, either class-by-class or together, were significantly higher overall than the corresponding maternal fecundities in the heterosexual sample. In contrast, none of the paternal female fecundities in the homosexual sample, either class-by-class or together, were different from the corresponding paternal females in the heterosexual group. The total fecundity of all female relatives of homosexuals was significantly higher than the total fecundity of all females in the heterosexual group, reflecting the greater contribution of maternal female fecundity. This pattern implicates the involvement of the X-chromosome in the genetic mechanism [60] and further suggests that sexually antagonistic selection via increasing maternal female fecundity significantly compensates for the reduced fecundity of homosexuals, thus resolving the evolutionary conundrum of the genetic stability of male homosexuality in the population, as previously suggested [6], [11], [18], [21], [34], [35], [36].

A question rises whether it is appropriate to test balancing selection hypotheses in low fertility populations, such as Italy and Spain. Perhaps these lower fertility populations might provide anomalous results compared with natural (high) fertility populations which might be more ideal to test balancing selection hypotheses. Camperio Ciani, Cermelli and Zanzotto [21], however, suggested in detailed mathematical notions that even if it seems counter-intuitive, the partial effect of genetic factors, influencing fecundity, increases with decreasing fecundity within populations (Figure 3 in Camperio Ciani et al. [21]), producing a “buffer effect”, which might explain the high frequency of male homosexuality in ancient Greeks, Romans and modern urban populations all with progressively declining fecundity [61]. A possible explanation is that while ecological factors, such as nutrition and social condition, largely influence the fecundity of a high fecundity population, in a low fecundity population, fecundity is primarily influenced through internal motivational factors that are potentially targeted through genetic differences. [21], [35] Thus, low fecundity populations present the best cases to identify potential genetic effects on fecundity and sexual orientation.

The sample investigated in this study (Table 1) was highly homogeneous in terms of the age of the homosexual and heterosexual probands, and most of the significant differences reflected the direct comparison of the raw data (Table 2, 3). However, we further controlled for age for several reasons. First, compared with other Western countries, Italy has experienced one of the highest steady, progressive fecundity declines over the last fifty years, potentially reflecting cultural or social aspects, which have led to a 50% decrease in fecundity from 3.6 to 1.54 offspring per female in 2009 [58], [62]. Women who were born earlier within this strong cohort trend were expected to be more fecund because they belonged to an earlier age cohort. Second, there was a systematic age difference between the paternal and maternal females due to the common southern European tradition of marrying a younger wife, approximately 4–5 years, depending on the époque; this effect was larger in the past century and has decreased only within recent years [59]. Due to the asymmetry in the age at marriage, the aunts and grandmothers of the paternal lines of the probands were approximately 5 years older on average than the aunts and grandmothers of their maternal lines. The age difference observed in our sample of aunts was consistent with the age difference at the time of marriage of couples reported between 1960 and 1980 [59]. Third, the average age of our homosexual sample was minimally, but significantly, lower than that of the heterosexual sample, and thus the cohort fecundity effect was also affected through the female relatives of homosexuals compared with female relatives of heterosexuals, albeit to a lesser extent. Fourth, our first sampling of the homosexual and heterosexual pedigrees was performed in 2002 and the last was performed in 2011; therefore, the first females sampled from both the homosexual and heterosexual probands were derived from an age (hence socio-cultural) cohort at ten years earlier. We controlled for these age effects using ANCOVA between and within our two samples.

The results (Table 4, 5) controlling for these age effects with ANCOVA confirmed and expanded the previous raw data results. The maternal line females of the homosexual group were significantly more fecund than the paternal line females, which was true for both aunts and grandmothers, whereas the paternal females did not show any significant differences between the homosexual and heterosexual groups. Once corrected for age effects, a comparison of the fecundity between the maternal and paternal lines within proband groups showed that both the maternal aunts and maternal grandmothers of homosexuals were more fecund that the paternal aunts and grandmothers of homosexuals, whereas the paternal aunts of heterosexuals were significantly more fecund than the maternal aunts of heterosexuals.

The significant increase corresponded selectively with the maternal aunts and grandmothers, and the higher fecundity of the maternal grandmothers together with the higher fecundity of maternal aunts was sufficient to significantly increase the overall fecundity of the female relatives of the homosexual probands. Our results were in contrast with the predictions of overdominance, which suggests that there should also be higher fecundity among the paternal female relatives of homosexuals [19], [20], [21], [32], [41], [42]. Remarkably, the paternal aunts exhibited a higher fecundity than the maternal aunts in the heterosexual group, as the hypothesis of sexually antagonistic selection predicted, although it was not immediately self-evident. [21] In a mathematical analysis of population dynamics, Camperio Ciani et al. [21] showed that under sexually antagonistic selection an increase of fecundity among the paternal female relatives of heterosexuals together with a higher fecundity of maternal females in the homosexual group is consistently observed [21]. This pattern was never considered based on previous empirical observations, as it was not obvious why the absence of an X-linked factor would also increase fecundity in the heterosexual paternal line. This increase might reflect the fact that more fecund females influencing homosexuality in males could be included with higher probability into the sample of heterosexuals only from the paternal side and, thus, increase this class of fecundity; notably if these females entered in the maternal side the proband would be more likely homosexual and the family would be classified as such. However this interpretation needs to be confirmed, as upon re-analyzing all of the previous data, increased fecundity was observed. The observed increased fecundity of paternal aunts compared with the maternal aunts of heterosexuals in the absence of the factor, further supports the theoretical predictions of the X-linked sexually antagonistic selection hypothesis [21]. While these results confirm the X-linked effect for homosexuality as Hamer initially proposed [7], [10] and the evolutionary conclusion of Camperio Ciani and colleagues [6], [10], [11], [13], [18], [20], [21], [34], [35], [43] based on a much larger data set, some discussion is necessary to address the relevant studies addressing this conundrum that did not reach the same conclusions.

### Balancing Selection and Fraternal Birth Order Effect

Camperio Ciani, Corna and Capiluppi [11] showed that mothers of homosexual men were significantly more fecund than the control-matched mothers of heterosexual men. Blanchard [55] noted that a research design based only on maternal fecundity was somewhat problematic because of the fraternal birth order, which could inflate the mean size of the sibships of the homosexual group. However, Camperio Ciani, Corna, and Capiluppi [11] maintained that a fecundity advantage was also present in the mothers of first-born homosexuals, for whom the fraternal birth order cannot be implicated. However, due to the small sample size of the initial study, their results were not highly significant. Later, Iemmola and Camperio Ciani [34] performed a study in a larger sample, which provided higher statistical significance, even in mothers of first-born homosexuals, suggesting that the balancing effect was accurate. Blanchard [55] confirmed the increased fecundity of mothers of first-born homosexuals through the analysis of four large sample sets of mothers of first-born homosexuals, showing that first-born homosexual mothers did not show a fecundity increase and suggesting that the fecundity increase could be an artifact of the fraternal birth order [55]. Riger et al. [41] promptly replied to Blanchard [55], arguing that in their large sample of homosexuals, the mothers of a first-born homosexual were indeed significantly more fecund than the mothers of heterosexuals, thus confirming the conclusions obtained in previous studies [11], [18], [21], [34]. The database assembled by Blanchard [55] was impressive and cannot be easily dismissed. However, several observations can be made. First, the age difference between homosexuals and heterosexuals in the previous analysis was large [55], showing a range of greater than 10 years in some samples, and included heterosexuals who were much younger than homosexuals. Thus, correcting for age in this case could result in particular distortions [63], [64]. Notably, the correction for age is necessary to control for the cohort effect, as mothers born earlier belong to a more fecund cohort if all other assumptions, such as the linearity of the variable regressed, are respected. However, in our species, the age distribution of female fecundity is different from that of males, which is linear from age 20 to 50. In contrast, among modern Western females, the fecundity distribution is highly skewed. The fertility peaks in women in their early 20s and considerably declines after the age of 35, becoming null before 50 [65]. Thus, a linear regression of age, as shown through ANCOVA, could artificially overestimate fecundity in younger mothers, with some residual fecundity that might be expressed, thereby distorting the ANCOVA and resulting in an overestimation of younger female fecundity; thus, an increase in the fecundity of the heterosexual sample would be observed in the Blanchard data. Because of this well-established and persistent problem with ANCOVA [63], [64], [66], [67], the raw data should be included to estimate the actual effect of age co-variation [68], as performed in the present study. An advantage of the present study is that it focuses only on the older female relatives of the probands, whose fecundity was definitively complete at the time of investigation.

A second observation refers to the inclusion [55] of a sample from a previous study [37] in the analysis of the fecundity of mothers with a first-born homosexual son [55]. However, the inclusion of the Blanchard and Lippa BBC Internet database [37] was problematic, as the authors observed that the fraternal birth order effect was weaker and more inconsistent than previous studies. Blanchard and Lippa suspected that the participants responded less conscientiously through the Internet than conventionally examined participants. Consequently, a series of filters and statistical operations was employed to remove inaccurate respondents and confounding effects. Furthermore, the probands in this Internet population study belonged to a population adopting a so-called “stopping rule”, which comprises individuals that continue having additional offspring until acquiring children of both sexes [69], [70]. The adherence to this common, but confounding rule obliged the authors to limit their results only to right-handed and non-last-born homosexuals to determine fraternal birth order effects. Approximately 20% of the sample was excluded due to unreliable answers based on incongruence between the reported family size and sibling composition. However, this exclusion could not be realized for the sub-sample size in the Internet study of the mothers of first-born homosexuals [55]. We hypothesize that a similar percentage of unreliable respondents might also be present among the first-born respondents. Unfortunately, this aspect cannot be controlled because cross checking was not possible for the first-born probands based on the limited questions included in the original study [37], [71]. Notably, the Blanchard and Lippa showed that whereas the fraternal birth order effect was highly complex and difficult to observe, the significantly higher number of overall offspring of the mothers of homosexuals, in accordance with the balancing selection hypotheses, was indeed evident [37].

### X-chromosome-linked Sexually Antagonistic Selection Versus Autosomal Overdominance

A further question that naturally arises is why other studies have not observed the increased female fecundity of the maternal line. King et al. (2005) and Schwartz et al. (2010) observed a generalized increase in fecundity [19], [32]. However King et al. provided some questionable results because the study did not analyze the specific fecundity of each gender class, but rather, described family size; thus, it is difficult to interpret the individual fecundity contribution to the increased family size [32]. Schwartz and colleagues [19] conducted a study that replicated the study of King and colleagues [32] with a larger sample collected from festivals and gay pride events in the U.S. However, because the results substantially replicated those of the previous study [32], the same questions emerged regarding their pedigree. As Riger et al. noted [41], the heterosexual sample that Schwartz et al. used showed a surprisingly low fecundity compared with similar samples, which might indicate sampling problems. Moreover, the collection method of Schwartz et al. was unclear with respect to consideration for the relative age of the relatives and the systematic age differences between the paternal and maternal relatives, as this information is relevant with respect to the considerably large 6-year difference between the homosexual and heterosexual samples in the Schwartz et al. study, which is unexplained through a demographic comparison specifically designed to assess the differential fecundity between probands and their relatives. The application of ANCOVA for this sample would reveal the previously discussed weaknesses of the covariance analyses controlling for age [63], [64], [66], [67]. Notwithstanding, the results of this study were enlightening. Although these authors argued against a prevalence of male homosexuality in the maternal line, the reanalyzed results from their published data (Table 2 [19]) showed a 5.07% enrichment of homosexuality in the maternal line versus a 2.61% enrichment in the paternal line (X^2^ = 72, df 1, p<0.0001), consistent with the sexually antagonistic hypothesis. This outcome can be derived by calculating the ratio of homosexuals among the maternal relatives sharing the X-chromosome with the proband (i.e., brothers, maternal uncles, and maternal cousins from aunts) and the ratio of homosexuals in the proband classes that did not share the X-chromosome (i.e., fathers, grandfathers, paternal uncles, paternal cousins of both aunts and uncles and maternal cousins from maternal uncles) from their published table.

Indeed, the data presented in Tables 2 and 3 by Schwartz et al. suggests a maternal increase in fecundity [19]. In Table 3, the presented fecundities are aggregated between males and females making it difficult to determine an X-chromosome selective fecundity effect. However, we were able to calculate the number of maternal aunts in the Schwartz et al. sample using the number of maternal uncles published in Table 2. By subtracting maternal uncles from the aggregated number of maternal uncles and aunts, the number of maternal aunts was determined as 1080. Using the same procedure, the number of paternal aunts was determined as 1065. Subsequently, from the published number of male cousins from maternal (n = 852) and paternal aunts (n = 696) published in Table 2 and considering that in human populations, the ratio of male live births to female live births is close to 106:100 [72], [73], we estimated the maternal aunt fecundity was calculated as 1.532 (1655/1080) offspring produced on average. In contrast, the fecundity of paternal aunts was 1.26 (1352/1065) offspring produced. Thus, these differences were highly significant (X^2^ = 56.2, df 1, p<0.0001). The same comparative analysis of the fecundity of the aunts of the heterosexual probands showed no significant difference (X^2^ = 2.6, df 1, p NS). Based on these calculations, the data of Schwartz et al. supports the hypothesis of sexually antagonistic selection based on empirical evidence [19]. Thus, we suggest that these data are not compatible with the overdominance hypothesis. Unfortunately, it was not possible to further test these hypotheses using the data presented in the tables from this study.

### Sexually Antagonistic Selection as a General Genetic Mechanism to Balance Male Homosexuality in our Species

The maternal line fecundity effects observed in the present study have been previously demonstrated in European (Italian, Spanish and French) samples [11], [18], [21], [35] in a large BBC Internet sampling of primarily Anglo-Saxons [37] and Samoan populations [38], [39], [74] in the United States (if our re-analysis of the previous data [19] is correct) and in an English Caucasian sample [36]. However, the Rahman study [36] is also widely cited because of its non-white sub-sample, where fecundity was shown to be higher in heterosexual probands. Based on these results, many authors suggested that fecundity asymmetries involving increased maternal line female fecundity are a local variation present only in Western or Caucasian homosexuals [19], [34], [35], [75]. The different white versus non-white patterns of fecundity suggested that it might be important to consider ancestry [19]. However, in this study a sample of non-white homosexuals is described, with non-trivial confusion, as comprising 20 probands, with mothers producing 88 offspring, exhibiting a mean fecundity of 4.4 offspring, which was much lower than that of non-white heterosexuals (53 proband, with mothers producing 731 offspring), exhibiting a surprisingly high average fecundity of 13.79 offspring (previously unrecorded and incomparable, even with maternal aunts of the same sample; Table 4 in Rahman et al. [36]). However, in Table 6, the same authors presented data from a sibling sex composition of non-white probands, and we could re-calculate maternal fecundity, summing the frequency of all sibling classes, including the respective number of probands, to obtain an average fecundity of 5.2 for mothers of non-white heterosexuals and a fecundity of 3 for mothers of non-white homosexuals, which is not consistent with the average published Table 4 and establishes further confusion. We conclude that until the data on additional non-Western populations are properly examined, the maternal line female fecundity increase should be accepted as a general occurrence irrespective of ethnicity.

### Limitations

This paper attempts to explain the persistence of genetic factors influencing male homosexuality through a Darwinian paradox, thereby giving the impression that sexual orientation is exclusively determined through genetic factors, which is not true. In a previous study using pedigrees and questionnaires, it has been suggested that genetic and biological influences on sexual orientation could explain approximately 20% of the variance in sexual orientation [11], suggesting that genetic factors do not determine sexual orientation in males; thus, important research has been conducted to understand the social and developmental factors that influence the remaining variation. [16].

Sampling homosexual or heterosexual probands and deriving population demographic information could generate some biases, such as recalling ages of specific subjects, which should be considered. Accessing a ‘hidden’ population is difficult because no sampling framework exists, and the public acknowledgement of membership could potentially be prejudicial to the subjects or a fraction thereof. Thus, standard probabilistic sampling methods produce low response rates and unreliable responses [76]. Targeted sampling is a widely employed method for accessing hidden populations, which is preferred to snowball sampling [77] and other examples of chain referral. These procedures introduce well-documented biases [78]. More generally, estimates referring to population units different from the survey units (i.e., the population of the grandmothers or aunts of the interviewed subjects) were not corrected through weighing the units with the inverse of their probability of selection in the sample (homosexuals), as previously suggested [78], [79], reflecting the fact that these estimates were compared with similarly collected samples (heterosexuals). The same methodology was used in the same locations to reproduce potential biases and increase the internal validity of the comparisons. We considered these biases to have limited relevance in this study, and our procedures were unlikely associated with such biases, as only demographic variables (such as the number and age of grandparents, cousins or aunts and uncles) were assessed.

This study did not collect data on male fecundity. It is believed that male fecundity it is not relevant in this analysis for two reasons. First, according to general Darwinian theory, fecundity-related decisions are biologically more inherent to females than males, and the variables influencing the variance of reproductive success are different in males than in females. Second, paternity is much harder to assess than maternity. However, we cannot exclude the possibility that the inclusion of accurate male reproductive success data in a future study might lead to different conclusions.

Additional data, particularly data obtained from non-industrialized high fecundity societies, are needed to further confirm that sexually antagonistic selection is a widespread phenomenon explaining the male homosexuality conundrum. These data should focus on fecundity patterns in which no confusion with the fraternal birth order effect can occur, such as among grandmothers and aunts of probands, and to a lesser extent, the number of younger siblings of homosexual probands. The aim of this population genetics study was to understand the Darwinian paradox of the genetic component of male homosexuality, and no effort has been made to identify these genetic factors. Thus, we acknowledge that currently no genes influencing homosexuality have been identified, neither on the X chromosome nor on autosomes [15]; however, in light of the present results, it would be more promising to determine the genetic factors influencing fecundity in females.

### Conclusions

The results of this study suggest that in homosexual males, the significant increase in the fecundity of the maternal line is not a by-product or an artifact of the fraternal birth order effect, but rather, is a coexisting factor potentially influencing homosexuality in males. In the families of homosexual individuals, the fecundity of maternal grandmothers and aunts significantly increases compared with the families of heterosexual individuals. This selective increase in the maternal line fecundity is sufficient to increase the total female fecundity of a family with homosexual individuals, even if the fecundity of the paternal females does not increase.

Regarding the genetic contribution to fecundity, we suggest that sexually antagonistic selection, rather than overdominance, is the balancing selection that potentially functions through X-linked genetic factors to compensate for the reduced fecundity of male homosexuals via increased maternal line female fecundity. The confirmation of these findings through future studies would resolve the long-standing Darwinian paradox associated with male homosexuality.
